# An overview of elastic waveguides with dynamic sub-structures

**DOI:** 10.1098/rsta.2021.0381

**Published:** 2022-11-28

**Authors:** Leonid Slepyan, Alexander B. Movchan

**Affiliations:** ^1^ School of Mechanical Engineering, Tel Aviv University, PO Box 39040, Ramat Aviv, 69978 Tel Aviv, Israel; ^2^ Department of Mathematical Sciences, University of Liverpool, Liverpool L69 7ZL, UK

**Keywords:** Floquet waves, dispersion, resonant wave, structured waveguide

## Abstract

The dynamic response of elastic waveguides is important for a wide range of applications that involve dispersive waves as well as wave localization. In particular, a case of special interest relates to waveguides subjected to moving loads. In the case where the elongated structure includes a sequence of built-in resonators, the range of resonance regimes may be extended accordingly. The present paper gives an overview of several mathematical formulations that connect Floquet theory to the dynamic response of multi-scale waveguides, which include inertial sub-structures subjected to external forces.

This article is part of the theme issue ‘Wave generation and transmission in multi-scale complex media and structured metamaterials (part 2)’.

## 1. Introduction

Conventionally referring to the inertial system of coordinates as the one where the momentum of a particle is conserved when external forces are absent, we note that Newton’s laws of motion are typically considered relative to such inertial systems. Would the terms ‘negative inertia’ or ‘negative mass’ make sense? Many would be tempted to say ‘no’, assuming the layman notion of a mass as a non-negative quantity which can be measured in an inertial system of coordinates. On the other hand, why would it be of interest to consider dynamics relative to non-inertial systems? This is a rhetorical question, and of course everything on planet Earth should be referred to in a non-inertial reference system, due to the gyroscopic planetary motion, with the Coriolis effect and the vortex-like motion of the air mass around low-pressure areas being well-observed examples.

More than 50 years ago, a model of an elastic waveguide with a dynamic sub-structure was introduced by Slepyan [1], who analysed a *strain wave* in an elastic solid connected to vibration-isolated masses, including the evolution of a wave in a cylindrical elastic shell with the attached dynamic *macro*-structure (modelled by uniformly distributed mass–spring oscillators).

More recently, Milton and Willis published an article [2] on modifications of Newton’s second law and linear continuum elastodynamics; this paper introduced a theory for an elastic medium with a dynamic structure, where the inertia is no longer characterized by a non-negative scalar mass density. In particular, for elastic systems with built-in resonator sub-structures, negative inertia can be achieved by introducing a phase shift in oscillations of a resonator relative to the background main system.

In the context of the Floquet–Bloch waves in periodic elastic systems, elastic structures with built-in resonators are shown to support standing waves as well as localized waveforms. Detailed analysis of several classes of such elastic systems has been conducted by Bigoni *et al.* [3], Bigoni & Movchan [4] and Mishuris *et al.* [5,6]. Waves in a complex waveguide are topical in a wide range of interdisciplinary applications. Examples include the electro-mechanical interaction of waves in biological systems studied by Engelbrecht *et al.* [7] and the modelling of ‘nerve pulse propagation’ studied by Peets *et al.* [8] in a microstructured waveguide. The transmission problem for waves in water and air has been discussed by Bok *et al.* [9] in the context of wave interaction with a microstructured interface.

Here, we give an overview of several configurations representing waveguides with multiple resonators, which constitute a dynamic sub-structure. In the simplest case, we consider one-dimensional waves forced by a moving–oscillating load and propagating in a uniform waveguide with a dynamic structure. The monograph [10] presents a comprehensive theory of waves in lattice systems containing advancing cracks and moving loads. We discuss the questions of spatial scaling and explain the term ‘multi-structure’ in the context of wave dynamics. The question of phase shifts within the system of resonators is of paramount importance, and several examples are considered here. Dynamic loading of multi-structures, and in particular moving loads, are very important in the context of wave dynamics and wave localization. Finally, we touch upon an interesting phenomenon of dynamic fracture—when a fault is advancing in a dynamic elastic lattice, the method of analysis differs substantially from that for continuous models.

## 2. The notions of multi-structure and microstructure

In mechanics, the terms ‘multi-structure’ and ‘microstructure’ are used in parameter-dependent formulations involving a small parameter, which characterizes an elementary cell of a periodic pattern or a particular constituent within a connected mechanical system. The mathematical notion of multi-structure was introduced by Ciarlet [11] in the context of connections between high-contrast elastic plates. It was further extended by Kozlov *et al.* [12] to parameter-dependent systems which consist of elements of different limit dimensions, and the mathematical theory of multi-structures was described with an emphasis on the analysis of fields around junctions between different constituents. When multiple parameters are introduced and the background solid includes many constituents of different scales, the term *microstructure* is often used. In the context of physics, terms such as microstructure and nanostructure reflect the scale of the structure, e.g. micrometre and nanometre, respectively. However, when the governing equations and additional boundary and initial conditions are written in normalized form with a small non-dimensional parameter being present, a wide class of specific physical configurations can be considered. In the context of homogenization theory, static microstructures which consist of elastic rods were analysed by Panasenko [13]. Two-dimensional periodic dynamic elastic structures with built-in resonators and macro-cells of complex geometries were analysed in the context of Floquet waves and their dispersion by Martinsson & Movchan [14].

The question of the connection between different scales is important in understanding how a process observed on one scale can be modelled at a different scale. Let the material properties, and hence the wave speeds, be the same in both scales. In this case, it is assumed that the scale relations for length and time are X=λx and T=λt with λ being a constant, 0<λ<∞, where (x,t) and (X,T) are the initial and new coordinate–time pairs, respectively.

A point-mass–spring lattice is a natural model of such a microstructure. On the other hand, the continuous, non-structural medium model provides a long-wave approximation (i.e. the wavelength is much greater than the lattice cell size).

Slepyan [1] considered the evolution of a wave in a cylindrical elastic shell with the attached dynamic *macro*-structure (modelled by uniformly distributed mass–spring oscillators). The same wave (with appropriately chosen time scale) can propagate in a continuous waveguide with microstructure.

There are physical examples where there is no such self-similarity. This happens when a quantity which is expected to be changing under the scale change is instead fixed. One example is the gravitational force: the acceleration under gravity (at the same height!) is constant. Surface energy of fixed value is another example.

## 3. Phase shifts for elastic waveguides with dynamic reflectors

To clarify the connection of the ‘negative inertia’ with phase shift in harmonic oscillations of multi-structures, we consider two illustrative one-dimensional examples.

In both examples, we assume that a sinusoidal wave propagates in a one-dimensional x-related waveguide, as follows:

3.1 $$u_{0}(t,x)=A_{0}\;e^{i(\omega t-kx)},\;\text{ where }A_{0}>0,$$

with speed v=ω/k>0. Let a resonance oscillator of eigenfrequency $\omega =\omega_{0}$ be attached to the waveguide at x=0. The oscillator is designed in such a way that the total field u(x,0) vanishes at x=0. Thinking of the wave reflection and transmission, when two scattered harmonic waves $u_{1-}$ and $u_{1+}$ of radian frequency ω propagate to the left and to the right as a result of interaction with the oscillator, we have the right wave $u_{1+}(t,x)=-u_{0}(t,x)$. Although the oscillator is under the resonant excitation, its amplitude will not grow to infinity but only until the moment when the sum $u_{1}(t,0)+u_{0}(t,0)$ becomes zero. At the same time, the oscillator’s action on the waveguide continues. The required scattered wave, $u_{1}(t,x)$, can be represented as

3.2 $$u_{1}(t,x)=u_{1+}(t,x)+u_{1-}(t,x),\;\text{with }\;u(t,0)=u_{0}(t,0)+u_{1}(t,0)=0,$$

where

3.3 $$\begin{array}{rl} & u_{1+}(t,x)=(-A_{0}\;e^{i(\omega t-kx)}+{\tilde{u}}_{1+})H(x)\\ \mathrm{and}\; & u_{1-}(t,x)=(-A_{0}\;e^{i(\omega t+kx)}+{\tilde{u}}_{1-})H(-x), \end{array}\}$$

with ${\tilde{u}}_{1\pm }$ being possible local disturbances such that

3.4 $$u_{1}(t,0)=\lim_{x\to \pm 0}u_{1\pm }(t,x).$$

As a result, we have the reflector defined as follows:

3.5 $$\begin{array}{rl} & u(t,x)=u_{0}(t,x)+u_{1+}(t,x)=0\;\text{when}\;x>0\\ \mathrm{and}\; & u(t,x)=u_{0}(t,x)+u_{1-}(t,x)\to 2iA_{0}\;e^{i\omega t}\sin(|kx|)\;\text{when}\;x<0. \end{array}\}$$

Now we shall discuss two physical examples which correspond to the above description.

### (a) Wave reflector in an elastic string

Vibrations of an elastic string subjected to a constant pre-tension and containing a spring–mass resonator attached at the origin are described by the inhomogeneous wave equation for the transverse displacement u(t,x):

3.6 $$\begin{array}{rl} & \varrho \frac{\partial^{2}}{\partial t^{2}}u(t,x)-T\frac{\partial^{2}}{\partial x^{2}}u(t,x)=P(t)\delta (x),\;P(t)=\varkappa (u_{0}(t)-u(t,0))\\ \mathrm{and}\; & m\frac{\;d^{2}}{\;dt^{2}}u_{0}(t)=-P(t), \end{array}\}$$

where u(t,x) and $u_{0}(t)$ are the string and oscillator displacements, P(t) is the force acting on the string at x=0, T and ϱ are the string tension force and the mass per unit length, and m and ϰ are the oscillator mass and stiffness.

We represent the string displacement as a sum of the incident wave satisfying the homogeneous string equation and the wave excited by force P,

3.7 $$u(t,x)=u_{i}(t,x)+u_{p}(t,x).$$

We take $u_{p}(t,x)$ to be the reflector,

3.8 $$u_{p}(t,x)=-u_{i}(t,x)=0\;(x>0).$$

So we define

3.9 $$\begin{array}{rl} & u_{i}(t,x)=A\;e^{i(\omega t-kx)},\;u_{p}(t,x)=-A\;e^{i(\omega t-kx)}H(x)-A\;e^{i(\omega t+kx)}H(-x),\\ & u_{p}(t,0)=-A\;e^{i\omega t},\;u(t,x)=0\;(x\geq 0),\\ & \frac{\partial u(t,-0)}{\partial x}=-2ikA\;e^{i\omega t},\;\text{where }A>0\text{ and }0<\omega =\sqrt{\frac{T}{\varrho }}\;k, \end{array}$$

and take the latter to be resonant,

3.10 $$\omega =\sqrt{\frac{T}{\varrho }}\;k=\sqrt{\frac{\varkappa }{m}}\;⟹\;k=\sqrt{\frac{\varkappa \varrho }{mT}}.$$

It follows that

3.11 $$\begin{array}{rl} & P(t)=2kTA\;e^{i(\omega t-\pi /2)},\;u_{0}(t)=\frac{P(t)}{\varkappa },\\ & \frac{\;d}{\;dt}u_{0}(t)=\frac{2\omega kT}{\varkappa }A\;e^{i\omega t}\;\text{and}\;u(t,0)=0. \end{array}\}$$

Thus, an oscillator of the wave frequency attached at a point is the dynamic reflector for a wave in the string.

### (b) The fourth-order dynamic reflector

Now consider a similar problem for a *bending beam* with a point oscillator attached. The equations are

3.12 $$\begin{array}{rl} & \varrho \frac{\partial^{2}}{\partial t^{2}}u(t,x)+D\frac{\partial^{4}}{\partial x^{4}}u(t,x)=P(t)\delta (x),\;P(t)=\varkappa (u_{0}(t)-u(t,0))\\ \mathrm{and}\; & m\frac{\;d^{2}}{\;dt^{2}}u_{0}(t)=-P(t), \end{array}\}$$

where D is the bending stiffness and the other notation is the same as in (3.6). This is a fourth-order problem, and the dispersion curve is a parabola defined by

3.13 $$\omega =\sqrt{\frac{D}{\varrho }}\;k^{2}.$$

As in the case of the string, the incident wave is

3.14 $$u_{i}=A\;e^{i(\omega t-kx)}.$$

However, the oscillator force, P(t), now induces two pairs of waves, the propagating ones and local (exponentially decreasing) disturbances,

3.15 $$u_{p}(t,x)=-A\;e^{i(\omega t-kx)}H(x)-A\;e^{i(\omega t+kx)}H(-x)+Be^{i\omega t-k|x|}\;\;\;(k>0).$$

The latter term serves as the continuation condition at x=0, from which the equality follows: $\partial u_{p}(t,\pm 0)/\partial x=0$. It gives us B=iA. The force-related discontinuity is

3.16 $$\frac{\partial^{3}}{\partial x^{3}}\;u_{p}(t,+0)-\frac{\partial^{3}}{\partial x^{3}}\;u_{p}(t,-0)=-4ik^{3}A\;e^{i\omega t}.$$

Thus,

3.17 $$\begin{array}{rl} & P(t)=-4ik^{3}DA\;e^{i\omega t}=4k^{3}DA\;e^{i(\omega t-\pi /2)},\;u(t,0)=-iA\;e^{i\omega t}\\ \mathrm{and}\; & \frac{\;d}{\;dt}u(t,0)=A\;e^{i\omega t}. \end{array}\}$$

Compared with §3a, in the case of a vibrating flexural beam, the dynamic reflector leaves an exponentially decreasing perturbed area behind.

At the same time, the energy flux in the incoming wave is completely reflected. It is clear that there are no period-averaged energy fluxes between the waveguide and oscillator (since in the steady-state regime considered, the oscillator average energy is unchanged). This fact is reflected by the −π/2 phase shift; see equations (3.11) and (3.17). The phase shift in forced oscillations and associated waves has been recently considered in [15].

## 4. Discrete–continuous structures subjected to dynamic loading

In the recent paper [15], forced waves were considered in a *master* body equipped with a dynamic sub-structure or in contact with an ambient medium.

The non-local integral relations connecting the forces on the one hand and the displacements on the other may be highly effective for solving problems involving moving loads. Of course, in this case, the corresponding Green’s kernels are also required.

As in [15], given R(t,x) as the fundamental solution for the structure, the displacement u(x,t) of the body and the force P(t,x) exerted on the body from the structure are related by the formula written in the convolution form

4.1 P(t,x)=−R(t,x) ∗∗ u(t,x),

where ∗∗ stands for double convolution with respect to x and with respect to t. In particular, if a point response of the structure to the attached solid is assumed and there are no additional spatial interconnections within the structure, then the convolution in x in (4.1) does not occur, and the equation becomes

4.2 $$P(t,x)=-R(t)*u(t,x)=\int_{0}^{t}R(t-\tau )u(\tau ,x)\;d\tau .$$

Using the notation L(t,x) for a linear operator of the problem describing the dynamics of an elastic solid, and assuming that Q(t,x) represents the external force moving with speed v and oscillating with radian frequency $\omega_{0}$, the integral form of the dynamic equation for the moving body subjected to both the structure response and a moving–oscillating external force can be written in the form

4.3 $$(L(t,x)+R(t,x))\;**\;u(t,x)=Q(t,x)=Q_{\eta }(\eta )\;e^{i\omega_{0}t},\;\text{ where }\eta =x-vt.$$

As demonstrated in [15], because the operator L contains an inertia term which takes into account the attached structure, the inertia of the connected elastic body includes an additional convolution term.

In this case, taking into account the modified inertia which accounts for the effect of the attached structure, the master body dynamics can be considered as a separate problem. It may resemble a modified Newton’s second law, also discussed by Milton & Willis in [2].

The role of wave dispersion in problems of elastodynamics of this kind is also highlighted in [15], in particular for an elastic string equipped with uniformly distributed oscillators with no interconnections between the latter.

### (a) An elementary example: dispersion of waves in an elastic string with attached oscillators

For the example of a scalar formulation describing waves in an elastic string containing additional oscillators, the derivation is straightforward. The governing equation for the double Laplace–Fourier transform $u^{LF}$ of the transverse displacement is

4.4 $$\left(\varrho s^{2}+Tk^{2}+\frac{\varkappa s^{2}}{s^{2}+\omega_{\mathrm{osc}}^{2}}\right)u^{LF}(s,k)=0,$$

where ϱ, T, ϰ and $\omega_{\text{osc}}$ are the string mass per unit length, the tension force, the oscillator’s spring stiffness and its frequency, respectively.

By considering the limit s→iω, the dispersion equation relating real k and ω can be written in the form

**Figure 1.**
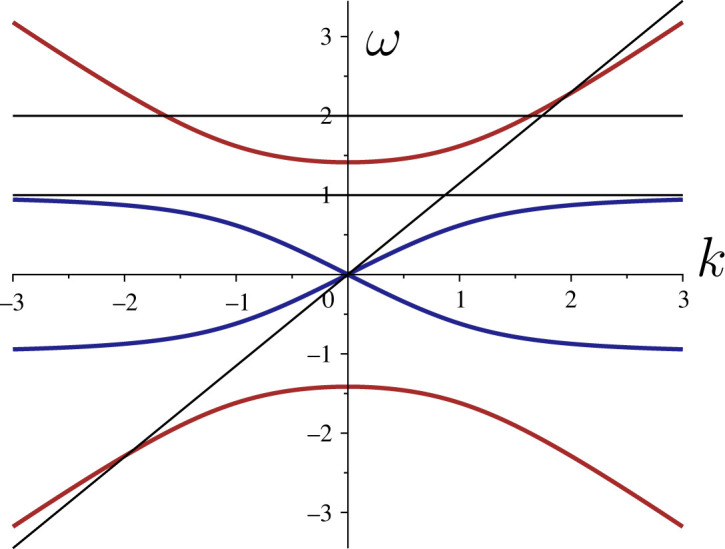
Dispersion diagram for waves in an elastic string with distributed oscillators. (Online version in colour.)

4.5 $$k^{2}-\frac{\omega^{2}}{c^{2}}=-\frac{\varkappa \omega^{2}}{T(\omega^{2}-\omega_{\mathrm{osc}}^{2})},$$

where the wave speed is defined by $c=\sqrt{T/\varrho }$. Figure 1 gives the graphical representation of the above dispersion relation in the non-dimensional form where the parameters ϰ,c,T and $\omega_{\mathrm{osc}}$ are all taken to be equal to unity. In this normalized form, the two branches correspond to the non-dimensional equation $(k^{2}-\omega^{2})(1-\omega^{2})=\omega^{2}$; they characterize the influence of the oscillator on the elastic string. The horizontal lines represent the oscillating loads, which do not move; for example, the lower line tangent to the lower branch is related to the resonant excitation. The inclined line corresponds to the moving (v>0) non-oscillating load.

### (b) Commentaries on the forced waves

A wave process associated with a moving force acting on an elastic solid with a microstructure is a transient process, which is challenging for analytical modelling. However, if the force is moving with a constant speed, rigorous analytical approaches have been developed in [10] and demonstrated for a variety of different physical configurations (see [1,10,15]). Often, the problem formulated in terms of integral transforms can also employ the dynamic Green’s function, which inevitably includes information about the dispersion properties of waves supported by the structured solid in question.

The solutions incorporate important information about the dynamic homogenization, as well as special cases which stand apart from the homogenization theory. In particular, these include description of the wavefront, as well as resonant cases, when the velocity of the applied load tends to the group velocity of the wave within the structure. Problems of such types have been analysed for both second-order equations for waves in forced structured strings and flexural elastic beams where the waves are governed by a fourth-order partial differential equation. The analytical approach provided by these solutions can be used effectively in numerical simulations and classification of the loading parameters, as discussed in [5,6,10], in problems where direct transient numerical simulations are extremely challenging and often inconclusive.

## 5. Dynamic failure and ‘knife waves’ in lattice systems

The idea of the modified inertia for the case of periodically distributed resonators in discrete or continuous systems also applies to problems of dynamic fracture. According to the general theory of [10], the advance of cracks in continuous elastic media is different from the propagation of faults in dynamic elastic lattices. The inertia of the nodal masses within the lattice gives rise to effects similar to those observed for structured waveguides, as discussed in §4. In particular, the use of lattice Green’s functions is of paramount importance. In a special case, when the advancing fault in the lattice is of semi-infinite extent, the mathematical approach is based on analysis of the functional equations of Wiener–Hopf type (see [10]).

The important phenomenon of wave localization has been studied in [16], where a mode III lattice with an interface layer was considered, with the dynamic crack advancing along the interface, aligned with the horizontal x-axis. In the dynamic fracture scenario, the ‘feeding wave’, which is also referred to as the ‘knife wave’, delivers the energy to the moving crack front, while the ‘dissipative waves’ carry a part of this energy away from the front.

In [16], the displacement $u_{m,n}(t)$ in the lattice at a nodal point (m,n) is sought in the form

5.1 $$u_{m,n}(t)=U_{n}(\eta ),\;\text{ with }\eta =m-vt,$$

where m and n are integers, t is time and v is the speed of the crack propagating along the interface. Although v is assumed to be constant, the Green’s function employed in the analysis is the dynamic lattice Green’s function, which takes into account the dispersion of the waves associated with the periodic lattice. While the structure is assumed to be symmetric with respect to the x-axis, the interface includes the central two horizontal layers (with nodal masses $M_{1}$ and spring stiffness $C_{1}$), and the ambient lattice has different inertial and elastic properties (nodal mass $M_{2}$ and spring stiffness $C_{2}$) from the interface itself.

To reduce the model to the analysis of a functional equation of Wiener–Hopf type, a Fourier transform is applied with respect to the horizontal variable η. In particular, the Fourier transform of the displacement in row n=1, representing the upper border of the interface, is written as

$$U_{1}^{F}(k)=U_{-}+U_{+}(k),$$

where

$$U_{+}(k)=\int_{0}^{\infty }U_{1}(\eta )\;e^{ik\eta }\;d\eta \;\text{and}\;U_{-}(k)=\int_{-\infty }^{0}U_{1}(\eta )\;e^{ik\eta }\;d\eta .$$

The corresponding Wiener–Hopf equation has the form

5.2 $$U_{+}(k)+L(k)U_{-}(k)=0,$$

with kernel function L(k) of the form

5.3 $$L(k)=\frac{P_{2}(k,0+ikv)}{P_{1}(k,0+ikv)},$$

where, for the case of $C_{1}=C_{2}=C$, the functions $P_{1}\text{ and }P_{2}$ are given by

$$P_{1}(k,i\omega )=\left(\frac{M_{2}}{2}-M_{1}\right)\frac{\omega^{2}}{C}+3-\cos(k)+\text{sgn}(\Omega )\sqrt{\Omega^{2}-1}$$

and

$$P_{2}(k,i\omega )=\left(\frac{M_{2}}{2}-M_{1}\right)\frac{\omega^{2}}{C}+1-\cos(k)+\text{sgn}(\Omega )\sqrt{\Omega^{2}-1}.$$

We note that the poles and zeros of the kernel function L provide information about the dispersion in the periodic lattice ahead of the crack and behind the crack within the lattice. Using the factorization

$$L(k)=L_{+}(k)L_{-}(k),$$

the factorized form of the Wiener–Hopf equation is

$$\frac{U_{+}(k)}{L_{+}(k)}+L_{-}(k)U_{-}(k)=0.$$

The solution of this Wiener–Hopf equation, analysed in [16], provides valuable insights into the relation between the feeding wave (knife wave) and the wave radiating from the moving crack. The work presented in [16] includes a study of the range of compatible crack speeds, and a detailed comparative analysis was given for this solution and a numerical simulation of the transient nonlinear model, where the advance of the crack through the lattice has a ‘staircase’ structure.

## 6. Concluding remarks

The term ‘homogenization’ is widely used in the engineering and mathematical literature, including studies of partial differential equations with rapidly oscillating coefficients. For problems of elastodynamics, homogenization has a completely different meaning than in static or quasi-static formulations. While homogenization models of elastostatics deliver important data about the elastic properties of the effective material, in elastodynamics one also needs equally important information about the inertia terms. In particular, in [2] it was demonstrated that characterization of the inertia by a scalar term involving a non-negative mass density may not be sufficient. Instead, inertia tensor quantities may be required to characterize the dynamic response of microstructured solids. The overview of structured waveguides presented here shows an alternative to classical homogenization approaches, especially in the case of moving–oscillating loads. In this context, general representations of the solutions involve Green’s kernels and convolution-type integrals, which take into account the structured nature of the waveguide. The term ‘microstructure’ is not needed in this case, since the approach does not depend on the structure scale.

The role of uniformly distributed and localized dynamic structures, which can also be referred to as oscillators, has been demonstrated in the context of wave dispersion, as illustrated for the simplest waveguides such as the elastic string and a flexural beam.

While considering the energy flux from the oscillating load to the waveguide, the phase shift appears to be important.

The ‘modified Newton’s law’ concept introduced in [2] has been explained here for a wider class of problems for multi-scale waveguides with built-in resonators, as well as moving loads.

Although some key points related to the waves associated with moving–oscillating loads have been discussed, this broad topic remains open. In this context, some interesting examples that involve three-dimensional wave localization were discussed in [6]. Studies of three-dimensional forced waves in structured solids remain vitally important, including the phase shifts and effects of negative inertia.

## Data Availability

This article has no additional data.
